# Hypocomplementemia as a Risk Factor for Organ Damage Accrual in Patients with Systemic Lupus Erythematosus

**DOI:** 10.1155/2018/8051972

**Published:** 2018-12-30

**Authors:** Warren Raymond, Gro Eilertsen, Johannes Nossent

**Affiliations:** ^1^Rheumatology Group, School of Medicine, University of Western Australia, Perth, Australia; ^2^Molecular Inflammation Research Group, Department of Clinical Medicine, Arctic University, Tromsø, Norway; ^3^Department of Rheumatology, Sir Charles Gairdner Hospital, Perth, Australia

## Abstract

While it is a common practice to monitor complement levels in patients with systemic lupus erythematosus to aid in flare prediction and detection, it is unclear if this strategy is helpful in preventing subsequent organ damage. We studied longitudinal complement levels in 102 SLE patients during a median follow-up of 13.8 years (IQR 7.0, 23.1). Low complement was defined as C3 < 0.84 g/L and/or C4 < 0.08 g/L, disease activity by clinical SLEDAI-2K, and organ damage by SLICC-DI. We calculated a time averaged clinical SLEDAI score (cWAS) and performed multivariate regression models to assess the independent predictive value of low complement for organ damage at last visit. Hypocomplementemia (HC) was observed in 67% of all patients and was more often due to low C3 (97%) than low C4 (54%). Compared to patients not developing HC (33%), HC patients were more frequently positive for anti-dsDNA Ab (72% vs 36%, *p* < 0.01) and aPL (74% vs 40%, *p* < 0.01) but HC was concurrently present with anti-dsDNA Ab in only half the cases. The time-adjusted cWAS scores (1.9 vs 1.2, *p* = 0.9), frequency (SDI > 0, *n* = 60), and type of organ damage accrual were similar for patients with and without HC (OR 1.08, *p* > 0.20). Intermittent or sustained HC has no predictive value for damage accrual in SLE or the underlying disease activity over time. This together with significant discrepancies in the concurrence of low C3, C4, and anti-dsDNA Ab indicates frequent activation of the complement pathway by other factors than immune complexes in SLE.

## 1. Introduction

In systemic lupus erythematosus (SLE), defective clearing of apoptotic material contributes to formation of autoantibodies and immune complexes (ICs). The complement system is an important host mechanism for the removal of atypical antigens and IC [1], and in SLE, hypocomplementemia (HC) is considered a serological sign of impending or ongoing inflammation where complement factors are “consumed” by tissue bound immune complexes (ICs). The severity and site of resulting clinical symptoms together with the frequent need for immunomodulating drug treatment underwrites the risk of organ damage accrual and premature mortality in SLE [2–4]. HC is included in the latest SLICC classification criteria as well as in disease activity scores for SLE (SLEDAI-2K) [5–9]. The reliability of HC as a serological reflection of underlying inflammation is uncertain as complement levels vary between healthy individuals [10–12] and complement synthesis decreases with liver disease and increases during infection, tissue damage, and hyperglycemia [2, 9, 12–19]. Furthermore, the specific development of anti-C1q Abs in SLE can dampen or increase complement consumption and together this may lead to normal complement levels during active disease [20–23]. Disease activity is strongly and causally associated with organ damage accrual, and the accrued amount of organ damage is the most prominent predictor of survival in SLE [24]. As there is limited data available, we investigated the role of HC as a risk factor for organ damage accrual in SLE.

## 2. Method

We performed a retrospective analysis of prospectively collected longitudinal data of SLE patients (Table 1). All participants met American College of Rheumatology classification criteria and were followed for a median 10.6 years (IQR 5.1, 17.8; range 0.3–23.9) with 2-4 routine medical appointments per annum. Data recorded included clinical and serological findings, autoantibody status, SLEDAI-2K score for disease activity, and SLICC-DI damage score [25]. We defined HC as C3 < 0.84 g/L and/or C4 < 0.08 g/L (by laser nephelometer) while anti-dsDNA Ab presence was defined as any anti-dsDNA Ab titer above cutoff (as assays changed during the study period). We calculated a clinical SLEDAI (cSLEDAI) score by excluding HC and anti-dsDNA Ab from overall SLEDAI and then computed a previously validated time-weighted average scores for clinical SLEDAI (cWAS) to standardize disease activity across visits with different time intervals [26]. Flares were classified as mild, moderate, or severe according to SELENA-SLEDAI flare index [27].

Quantitative variables are described as frequencies and percentages or median and interquartile range (IQR). Comparative statistics included the chi-square tests and Mann-Whitney *U* test and Spearman correlation coefficients (Rs). Complement levels were measured approximately twice a year (median time between measurements was 0.59 years) (IQR 0.15, 1.55). We therefore performed two separate analyses of HC: one by defining HC as ever/never and one by defining HC as episodic (present in <2 subsequent visits) or chronic, i.e., present during ≥2 subsequent visits (equating a one year). As both analyses produced essentially similar results (available on request), we present results for HC ever only. HC was analyzed as a binary (ever/never present) and as a continuous predictor (number of times HC occurred) in age and length of follow-up-adjusted logistic and time-dependent Cox regression models using SDI > 0 as the dependent variable. All participants provided written informed consent following approval from the Regional Norwegian Research Ethics Committee (approval number: REK 2015/1400).

## 3. Results

A third of SLE patients (*n* = 33.3%) sustained normal complement (NC) levels across 387 clinic visits during 7.8 years (IQR 3.1, 14.8) follow-up (Table 1). Patients who developed HC (*n* = 69, 68%) were younger at disease onset (31.0 vs 43.7 years, *p* < 0.001), more often female (91% vs 79%, *p* = 0.06), and had lower waist circumference (69.3 vs 74.2 cm, *p* = 0.048). Low C3 was significantly more frequent than low C4 (97% vs 52%, *p* < 0.01), while simultaneously low levels were seen in only 48% of HC episodes (Table 1). Compared to NC patients, HC patients had a higher ever prevalence of anti-dsDNA Abs (72.5% vs 36.4%, *p* < 0.01) and aPL Abs (73.9% vs 39.4%, *p* = 0.001) but a similar rate of anti-ENA antibodies: SS-A (51% vs 47%, *p* = 0.67), SS-B (21% vs 16%, *p* = 0.53), RNP (33% vs 30%, *p* = 0.80), and Sm (27% vs 23%, *p* = 0.68). Also, the prevalence of anti-C1q (9% vs 17%, *p* = 0.34), antiribosomal-P (13% vs 11%, *p* = 0.83), or positive Coombs test (16% vs 27%, *p* = 0.24) was comparable. HC patients were more often prescribed cytotoxic drugs (50.0% vs 24.2%, *p* = 0.014) and prednisone (91% vs 71%, *p* = 0.004) (Supplementary Table 1).

The overall annual flare rate for the SLE cohort was 1.00 (IQR 0.50, 1.75) for mild and 0.24 (IQR 0.10, 0.64) for severe flares, and while all NC and HC patients experienced flares, more HC patients experienced a severe flare (94% vs 73%) (Table 2). However, the time averaged disease activity for the whole disease course was similar for the NC and HC groups with no association with thrombotic or obstetric APS for HC patients (Table 2).

The frequency of any organ damage accrual (SDI > 0, *p* = 0.910) or the development of severe damage (SDI > 3, *p* = 0.94) and amount of organ specific damage accrual were all similar across NC and HC patients (Table 3) as was SDI score at last observation (median 2, IQR 1-3, *p* = 0.9). The predictive value by logistic and Cox regression modelling for HC for SDI > 0 (OR 1.03) and for HC in combination with anti-dsDNA Ab for renal damage (OR 4.85) was no longer significant after adjustment for age and length of follow-up (Table 4 and Supplementary Table 2).

## 4. Discussion

A third of all patients in this otherwise representative SLE cohort never demonstrated evidence of hypocomplementemia, which is in line with a reported range of 30-50% patients maintaining normal C3 and C4 levels throughout their disease [9, 28–30]. NC patients in this study were older at SLE diagnosis by ~10 years and had increased waist circumference, which might suggest that increasing age and/or body mass index (BMI) provide protection against overt HC, e.g., by contributing to increased complement synthesis which upholds serum complement levels even in the face of disease activity [9, 28, 29, 31, 32].

All patients experienced mild disease flares approximately once a year and severe flares every three to four years with flares in HC patients skewed towards higher cSLEDAI scores due CNS and renal involvement. Despite this, the similar cWAS scores and flare rates suggest that the burden of disease activity over time was similar for HC and NC patients. Some methodological differences aside, this is in agreement with data from studies by Buyon el al. and Ho et al. showing no association between low C3 or C4 and flares, while Ramos-Casals et al. reported equal cumulative flares rates for HC and NC patients [9, 29, 33]. This lack of association between HC and disease activity (flares) may reflect the fact that not all SLE manifestations are immune complex mediated (Table 2) and suggest that while C3 and C4 monitoring in general is probably not advantageous or cost-effective in SLE as a predictor of damage accrual, it may potentially be clinically useful in a selected group of patients where significant prior disease activity has occurred in the context of HC [9, 29, 33–35].

Our study is one of the first to establish that HC is not a useful predictor of the risk for cumulative or site-specific organ damage development with multivariate analysis quantifying the risk of damage accrual attributable to HC to no more than 3-5% over 15 years of follow-up. Gandino et al. reported similar findings and were unable to link HC (fluctuant or persistent) or the presence of anti-dsDNA Ab to organ damage during follow-up [28]. Even the clinically feared combined presence of HC and anti-dsDNA Ab was not a significant risk factor for organ-specific renal damage after age adjustment (OR 1.84, *p* > 0.20). Together, these findings suggest that much of the damage development in SLE patients is dependent on mechanisms other than complement activation. As NC patients received less cytotoxic and corticosteroid agents in this study but nonetheless developed similar SDI scores, we can also theorize that in this subgroup where disease activity was not associated with manifest HC, treatment may not have been sufficient to prevent damage accrual.

There was an interesting discrepancy between finding low C3 (97.1%) and low C4 (52.2%) in this SLE cohort, and while also observed by others, this remains largely unexplained [9, 28]. Low C3 in the face of normal C4 levels does not fit well with classical IC-induced complement activation and anti-dsDNA Ab, which are considered the key antibodies for complement activation in SLE were simultaneously present with low C3 in only a third of all cases. As we also found no role for anti-ENA Ab and anti-C1q Ab in the risk of developing HC, this supports the assumption that in a significant proportion of SLE patients, alternate nonimmune complex-dependent complement pathway activation occurs quite frequently [36–39]. Recent experimental findings support a distinctive role for alternative pathway activation in human SLE with membrane attack complex (MAC) formation occurring in excess of the typical response to foreign pathogens [40, 41]. Although not routinely performed, including properdin and factor B in serological assessment could help delineate alternate pathway activation in SLE [19, 40, 42–44]. We found that HC patients more often carried aCL Ab as also reported by Ramos-Casals et al. [9] but found no association for HC with thrombotic/obstetric APS or vascular damage. This is in line with findings by Clowse et al. who found no difference in aPL Ab prevalence or pregnancy outcomes across complement status (NC vs HC) during pregnancy [45]. Finally, the presence of anti-dsDNA Ab in a large proportion of NC patients confirms that a significant amount of anti-dsDNA Ab lacks sufficient complement fixing ability, and if at all involved in clinical manifestations in NC patients, they must do so by pathways not involving complement [46, 47].

The limitations of this study should be kept in mind. Firstly, visit frequency was based on the clinical need for rheumatological consultation whereby patients who achieved and maintained disease quiescence had less frequent visits. However, our follow-up routine was in line with current recommendations [48], and we adjusted for this potential limitation by utilizing time-adjusted weighted averages of SLEDAI scores and risk quantification for HC. Our routine measurement of complement proteins follows a common clinical practice, but did not include activation products for C3 and C4, which in some studies have shown better correlation with especially lupus nephritis [49]. Similarly, we did not measure activation of alternative pathway activation, which can directly activate C3 convertase in the face of normal C4 levels. Finally, the homogeneity and clinical settings of this cohort limit the generalizability of the findings to populations with a greater degree of diversity. The strength of this study lies in the long observation period with a complete dataset for included patients.

## 5. Conclusions

Hypocomplementemia is unrelated to organ damage accrual in SLE patients. Discrepancies in the concurrence of low C3 and C4, disease activity, and anti-dsDNA Ab suggest that complement activation in SLE often occurs through pathways not involving immune complexes.

## Figures and Tables

**Table 1:** Demographic and serological descriptors of SLE patients with and without hypocomplementemia (HC).

	NC (*n* = 33)	HC (*n* = 69)	*p* value
Male	7 (21.2%)	6 (8.7%)	0.076
Female	26 (78.8%)	63 (91.3%)	
Age at first visit	43.7 ± 13.7	31.0 ± 12.6	<0.001
Diagnostic delay (years)	2 (IQR 0, 5)	1 (IQR 0, 4)	0.494
Follow-up in years	7 (IQR 3, 13)	12 (IQR 6, 22)	0.017
Smoking ever	20 (66.7%)	39 (59.1%)	0.480
Years smoking	20 (IQR 15, 30)	20 (IQR 13, 26)	0.886
Waist circumference	74.2 ± 13.6	69.3 ± 13.6	0.048
Anti-dsDNA pos. ever	12 (36.4)	50 (72.5)	<0.001
Antiphospholipid Ab	13 (39.4)	51 (73.9)	0.001
Lupus anticoagulant pos.	2 (6.1)	12 (17.4)	0.114
aCL-IgG	12 (36.4)	42 (60.9)	0.020
aCL-IgM	8 (24.2)	32 (46.4)	0.032
Low C3 or C4 ever	—	69 (100)	—
Low C3 + C4 ever	—	33 (47.8)	—
Low C3 ever	—	67 (97.1)	—
No. of low C3 episodes	—	8 (IQR 4, 14)	<0.001
Low C4 ever	—	36 (52.2)	—
No. of low C4 episodes	—	4 (IQR 2, 9)	<0.001

Figures indicate median with interquartile range or numbers (%).

**Table 2:** Comparisons of disease activity measures between SLE patient with normal (NC) and low complement levels (HC).

	NC (*n* = 33)	HC (*n* = 69)	*p* value
WAcS	1.2 (0.65, 3.22)	1.9 (0.9, 3.3)	0.91
Clinical SLEDAI max	8.0 (4.0, 12.0)	13.0 (9.0, 17.0)	<0.001
Mild flares (%)	33 (100)	69 (100)	—
Severe flares (%)	24 (72.7)	65 (94.2)	<0.001
Mild flares per annum	0.8 (0.4, 1.7)	1.1 (0.5, 1.85)	0.12
Severe flares per annum	0.2 (0, 0.5)	0.3 (0.1, 0.8)	0.010
Manifestation			
Convulsions	0 (0)	10 (14.5)	0.02
Psychosis	0 (0)	3 (4.3)	0.22
Retinal	1 (3)	1 (1.4)	0.59
Cranial nerve	1 (3)	6 (8.7)	0.31
Vasculitis	6 (18.2)	24 (34.8)	0.07
Cylindruria	4 (12.1)	19 (27.5)	0.06
Proteinuria	6 (18.2)	34 (49.3)	<0.001
Arthritis	25 (75.8)	48 (69.6)	0.346
Rash	19 (5.6)	59 (85.5)	0.002
Alopecia	12 (3.4)	43 (62.3)	0.014
Ulcers	8 (24.2)	36 (52.2)	0.008
Serositis	5 (12.1)	25 (23.2)	0.16
Fevers	6 (18.2)	31 (44.9)	0.008
Hemolysis	2 (6.1)	4 (5.8)	0.98
Thrombocytopenia	4 (12.1)	25 (36.2)	0.012
Leucopenia	6 (18.2)	32 (46.4)	0.003
Thrombosis	4 (12.1)	8 (11.6)	0.89
Obstetric APS	4 (12.1)	12 (17.4)	0.49

Figures indicate median with interquartile range or numbers (%). WAcS: weighted average clinical SLEDAI score (see Methods). Clinical SLEDAI max: highest cSLEDAI score observed during the disease course; APS: antiphospholipid syndrome.

**Table 3:** Comparisons of frequency and severity of damage accrual by SLICC damage index (SDI) between SLE patient with normal (NC) and low complement levels (HC).

SDI feature	NC (*n* = 33)		HC ever (*n* = 69)		*p* value
	*N* (%)	Median (CI)	*N* (%)	Median (CI)	
Any damage	19 (57.6)		41 (59.4)		0.91
Final SDI score		2 (1, 3)		2 (1, 3)	0.82
Final SDI					
0	14 (42.4)		28 (40.6)		0.94
1-3	15 (45.5)		31 (44.9)		
> 3	4 (12.1)		10 (14.5)		
Organ damage site					
Eye	2 (6.1)	1 (1, 1)	3 (4.3)	1 (1, 1)	0.13
Neurological	4 (12.1)	2 (2, 3)	15 (21.7)	1 (1, 2)	0.45
Renal	1 (3.0)	1 (1, 1)	9 (13.0)	1 (1, 2)	0.48
Pulmonary	3 (9.1)	1 (1, 1)	4 (5.8)	1 (1, 1)	0.91
Heart	6 (18.2)	1 (1, 1)	9 (13.0)	1 (1, 2)	0.31
Peripheral vascular	3 (9.1)	1 (1, 1)	4 (5.8)	1 (1, 2)	0.94
MSK	6 (18.2)	1 (1, 1)	15 (21.7)	1 (1, 2)	0.80
Skin	2 (6.1)	1.5 (1, 2)	1 (1.4)	2 (2, 2)	0.24
Gonadal	1 (3.0)	1 (1, 1)	3 (4.3)	1 (1, 1)	0.38
Endocrine	0 (0.0)	0 (0, 0)	1 (1.4)	1 (1, 1)	0.50
Malignancy	6 (18.2)	1 (1, 2)	7 (10.1)	1 (1, 1)	0.98

**Table 4:** Multivariate analysis of hypocomplementemia (HC) and anti-dsDNA Ab presence as a risk factor for SLICC-DI >0 at last visit by logistic (yes/no) and time dependent Cox regression (risk increase per episode of HC).

Risk factor	HC	HC + anti-dsDNA
Logistic regression	OR (CI)	OR (CI)
Unadjusted binary exposure	2.03 (0.78, 5.27)	4.42 (1.25, 15.64)
Unadjusted continuous exposure	1.07 (1.00, 1.14)	1.06 (0.98, 1.15)
Cox regression	OR (CI)	OR (CI)
Unadjusted binary exposure	2.06 (1.13, 3.74)	1.53 (0.82, 2.87)
Unadjusted continuous exposure	0.98 (0.95, 1.01)	0.99 (0.95, 1.03)
Age-adjusted binary exposure	2.38 (1.31, 4.35)	2.04 (1.05, 3.98)
Age-adjusted continuous exposure	0.97 (0.94, 1.00)	0.98 (0.94, 1.02)

Figures indicate odds ratios (ORs) with 95% CI.

## Data Availability

The datasets used and/or analyzed during the current study contain identifiable patient information and therefore are not publicly available. The corresponding author will respond to any queries about the data used in this study on request.

## Supplementary Materials

Supplementary Materials Supplementary Table 1: this table describes the relation between patterns of hypocomplementemia (HC) and use of immune-modulating medication in the SLE cohort. Figures given indicate odds ratios (ORs) with 95% CI. Supplementary Table 2: this table describes the results of a multivariate analysis of isolated hypocomplementemia (HC) and anti-dsDNA Ab presence as well as their concurrent presence as predictors of CNS and renal damage by logistic and Cox regression. Figures given indicate odds ratios (ORs) with 95% CI.
