# Supplementary material for: Hypocomplementemia as a Risk Factor for Organ Damage Accrual in Patients with Systemic Lupus Erythematosus
Source: J Immunol Res. 2018 Dec 30;2018:8051972. doi: 10.1155/2018/8051972 (PMC6330819; doi:10.1155/2018/8051972)
Supplement: Supplementary Materials — Supplementary Table 1: this table describes the relation between patterns of hypocomplementemia (HC) and use of immune-modulating medication in the SLE cohort. Figures given indicate odds ratios (ORs) with 95% CI. Supplementary Table 2: this table describes the results of a multivariate analysis of isolated hypocomplementemia (HC) and anti-dsDNA Ab presence as well as their concurrent presence as predictors of CNS and renal damage by logistic and Cox regression. Figures given indicate odds ratios (ORs) with 95% CI. [file 8051972.f1.docx]

Supplementary Table 1 This table describes the relation between patterns of hypocomplementemia (HC) and use of immune-modulating medication in the SLE cohort. Figures given indicate Odds ratios (OR) with 95% CI

Supplementary Table 2 This table describes the results of a multivariate analysis of isolated hypocomplementemia (HC) and anti-dsDNA Ab presence as well as their concurrent presence as predictors of CNS and renal damage by logistic and Cox regression. Figures Figures given indicate Odds ratios (OR) with 95% CI

Supplementary Table 1 Relation between patterns of hypocomplementemia (HC) and medication in SLE patients.

|  | NC (n=33) | HC (n=69) | p-value |
| --- | --- | --- | --- |
| Prednisone use | 21 (64%) | 58 (84%) | 0.04 |
| Daily Prednisone Dose | 8.4(IQR 5.0, 4.6) | 10.4(IQR7.0, 16) | 0.25 |
| Time averaged Predn/day | 4.6 (IQR 2.2, 7.0) | 4.6 (IQR3.1, 8.4) | 0.58 |
| Methylprednisolone iv ever | 2 (6.1%) | 6 (8.8%) | 0.63 |
| Hydroxychloroquine ever | 20 (60.6%) | 45 (66.2%) | 0.25 |
| Immunosuppressants | 7 (21.2%) | 29 (42.6%) | 0.01 |
| NSAID | 7 (21.2%) | 19 (27.5%) | 0.45 |
| Antihypertensives | 12 (36.4%) | 27 (39.7%) | 0.60 |
| Statins | 7 (21.2%) | 7 (10.3%) | 0.15 |
| Bone sparing treatments | 2 (6.1%) | 12 (17.6%) | 0.10 |
| Antidepressants | 3 (9.1%) | 4 (5.9%) | 0.58 |
| Anticoagulants / Antiplatelets | 14 (42.4%) | 30 (44.1%) | 0.59 |

Immunosuppressants: Azathioprine, Mycophenolate Mofetil, Methotrexate

Supplementary Table 2 Multivariate analysis of hypocomplementemia (HC) and anti-dsDNA Ab presence as a predictor of CNS and renal damage by logistic and Cox regression. Figures indicate Odds ratios (OR) with 95% CI

| **Neurological Damage** | HC | Anti-dsDNA | HC + Anti-dsDNA |
| --- | --- | --- | --- |
| Logistic regression |  |  |  |
| Unadjusted Binary Exposure | 2.01 (0.61, 6.63) | 1.13 (0.40, 3.17) | 2.90 (0.77, 10.95) |
| Unadjusted Continuous Exposure | 1.08 (0.99, 1.17) | 0.99 (0.89, 1.11) | 1.00 (0.94, 1.07) |
| Cox Regression |  |  |  |
| Unadjusted Binary Exposure | 2.09 (0.69, 6.33) | 1.27 (0.50, 3.25) | 1.22 (0.36, 4.13) |
| Unadjusted Continuous Exposure | 1.04 (1.00, 1.08) | 1.00 (0.95, 1.06) | 0.98 (0.90, 1.05) |
| Age-adjusted Binary Exposure | 1.18 (0.36, 3.86) | 1.62 (0.61, 4.30) | 1.22 (0.35, 4.23) |
| Age-adjusted Continuous Exposure | 1.01 (0.97, 1.06) | 1.00 (0.95, 1.05) | 0.98 (0.90, 1.05) |
| **Renal Damage** | HC | Anti-dsDNA | HC + Anti-dsDNA |
| Logistic regression |  |  |  |
| Unadjusted Binary Exposure | 4.80 (0.58, 39.5) | 1.57 (0.38, 6.46) | 10.67 (1.22, 93.0) |
| Unadjusted Continuous Exposure | 1.08 (0.97, 1.20) | 1.14 (1.02, 1.28) | 1.08 (1.01, 1.16) |
| Cox Regression |  |  |  |
| Unadjusted Binary Exposure | 4.95 (0.63, 39.22) | 1.70 (0.44, 6.60) | 4.85 (0.59, 39.8) |
| Unadjusted Continuous Exposure | 1.05 (0.99, 1.10) | 1.08 (1.02, 1.13) | 1.065 (0.998, 1.1) |
| Age-adjusted Binary Exposure | 2.77 (0.33, 23.20) | 1.37 (0.34, 5.45) | 1.84 (0.19, 17.5) |
| Age-adjusted Continuous Exposure) | 1.01 (0.95, 1.06) | 1.05 (1.00, 1.11) | 1.02 (0.95, 1.10) |
